# Editorial: Omics for the objective diagnosis and management of immune-mediated rheumatic diseases

**DOI:** 10.3389/fmed.2023.1127430

**Published:** 2023-01-13

**Authors:** Tieh-Cheng Fu, Yen-Ying Kung, Jr-Rung Lin, Ching-Mao Chang

**Affiliations:** ^1^Department of Physical Medicine and Rehabilitation, Chang Gung Memorial Hospital, Keelung, Taiwan; ^2^Division of Cardiology, Department of Internal Medicine, Heart Failure Research Center, Chang Gung Memorial Hospital, Keelung, Taiwan; ^3^School of Medicine, College of Medicine, Chang Gung University, Taoyuan, Taiwan; ^4^Center for Traditional Medicine, Taipei Veterans General Hospital, Taipei, Taiwan; ^5^Faculty of Medicine, National Yang Ming Chiao Tung University, Taipei, Taiwan; ^6^Institute of Traditional Medicine, National Yang Ming Chiao Tung University, Taipei, Taiwan; ^7^Clinical Informatics and Medical Statistics Research Center, Graduate Institute of Clinical Medical, Chang Gung University, Taoyuan, Taiwan

**Keywords:** genomics, proteomics, microbiota, transcriptomics, objective diagnosis and management

Autoimmune rheumatic diseases (ARD) such as systemic lupus erythematosus (SLE), Sjögren's syndrome (SS), rheumatoid arthritis (RA), systemic sclerosis, and vasculitis are chronic inflammation problems with mechanisms including oxidative stress, B/T cell imbalances, and type I interferon (IFN)-α, as well as some inflammatory mediators (1, 2). Diagnosing ARDs is complex, in part due to the high likelihood of confusing them with other diseases (3, 4). They usually are treated with glucocorticoid disease-modifying agents such as Hydroxychloroquine, Sulfasalazine, Methotrexate, and Azathioprine, or biologic agents such as Tumor necrosis factor (TNF) inhibitors (etanercept, infliximab, adalimumab, certolizumab pegol, and golimumab), Interleukin (IL)-1 inhibitor (anakinra), IL-6 inhibitors (tocilizumab and sarilumab) IL-17 inhibitor (secukinumab), T-cell inhibitor (abatacept), B-cell inhibitor (rituximab), or Janus kinase inhibitors (tofacitinib, baricitinib, and upadacitinib) (5). However, these drugs have low efficacy, relatively severe adverse side effects, or are very expensive (6–8).

Many recent studies have used “omics” tools such as genomics (9), transcriptomics (10), proteomics (11), metabolomics (12), and microbiomics (13) to approach ARD diagnosis and management objectively. Due to the complexity of the information involved, multi-omics analysis has been recommended as providing a more comprehensive view of these diseases (14–18). Through such integrative multi-omics analysis, it is widely expected that individual treatment will benefit from precision medicine with novel targets and treatment strategies (19–21).

Wang et al. who applied gut microbiomics to 19 SLE patients and 19 of their healthy family members, linked gut microbiota with 16S rRNA and SLE clinical manifestations. In particular, their results indicated lower alpha diversity and higher heterogeneity in the SLE group than the control group, which decreased the former group's *Acidobacteria, Gemmatimonadetes, Nitrospirae* and *Planctomycetes* at the phylum level, and increased *Streptococcus, Veillonella, Clostridium XI*, and *Rothia* at the genus level. Most importantly, *Streptococcus* was extremely enriched among these lupus patients. The operational taxonomic units of *Lachnospiracea incertae sedis* and *Parasutterella* were negatively correlated with decreases in blood platelets and pulmonary-artery pressure, respectively. However, the samples of SLE patients and healthy individuals in this study were both small, and its results should therefore be confirmed through large-scale investigation.

Because SLE and SS appear to share similar pathogenesis, understanding one of these diseases could help us to understand the other. Xiao et al. reported that N6-methyladenosine (m6A) RNA modification was connected with the pathogenesis of primary SS, based on a study that included 44 primary SS patients, 50 age-gender-matched healthy controls, and 11 age-gender-matched patients with non-SS sicca. Specifically, Xiao et al. measured the mRNA levels of m6A elements, ISG15, and USP18 in peripheral blood mononuclear cells (PBMCs), and found that the expression of METTL3, RBM15, ALKBH5, FTO, YTHDF1, YTHDF2, YTHDF3, YTHDC1, and YTHDC2 was higher in the primary SS patients than in the healthy controls. Additionally, the expression of METTL3, RBM15, ALKBH5, FTO, YTHDF1, YTHDF2, YTHDF3, YTHDC1, and YTHDC2 was higher in primary SS patients than in those with non-SS sicca. As such, it is possible that FTO, YTHDF2, YTHDF3, and/or YTHDC2 might target ISG15, and activate the type I IFN-signaling pathway in primary SS. It is worth mentioning that USP18 is also a member of the deubiquitinating protease family of enzymes, and it could inhibit IFN-α-mediated Jak/STAT signaling pathway (22, 23). Importantly, however, Xiao et al. only extracted mRNA from PBMCs. Detection of mRNA levels from salivary glands could be more direct and therefore more suitable for future studies of the same topic.

Not all RA patients present rheumatoid factor (RF) and anti-citrullinated protein antibodies (ACPA), and this complicates clinical diagnosis of RA. Ruiz-Romero et al. used iTRAQ-based quantitative proteomic analysis on 80 RA serum samples divided into four pools, verified the results with another 80 such samples, and validated them with 260 more such samples. Their findings suggested (1) that the serum levels of alpha-1-acid glycoprotein 1 (A1AG1), haptoglobin (HPT), and retinol-binding protein 4 (RET4) were higher in the double-seropositive group; and (2) that the increased level of A1AG1 was associated with RF (RF+A1AG+), whereas HPT was associated with ACPA (ACPA+HPT+). However, Ruiz-Romero et al. did not report their subjects' treatments, disease durations, disease activity, or possible extra-articular manifestations, all of which could be important predictors of RA.

T-cell large-granular lymphocytic (T-LGL) leukemia is associated with ARDs, especially RA. Gorodetskiy et al. enrolled 72 ARD patients with absolute T-LGL counts of <1.5 × 10^9/*L*^, and 11 ARD patients with absolute T-LGL counts <1.5 × 10^9/*L*^. The results showed the mutations in *STAT3* were detected in 56% of the 25 patients of monoclonal T-cell receptor gene rearrangement pattern, and the authors suggested that those patients who did not exhibit malignant LGLs in their blood or bone marrow had a “splenic variant” of T-LGL leukemia. However, such results still should be confirmed by large-scale investigation, especially among patients with no blood or bone-marrow involvement of malignant LGLs.

With the aim of accelerating objective diagnosis of ARDs, this special topic surveys the omics aspects of ARDs and how particular omics and their combinations relate to diagnosis and treatment. Identifying the association between genotypes and phenotypes will aid more accurate and efficient diagnoses by researchers and clinicians; and multi-omics analysis of molecular regulatory mechanisms and the roles of oral-gut microbiota in ARDs can be expected to help researchers and clinicians find novel targets for treatment.

## Author contributions

T-CF, Y-YK, J-RL, and C-MC were responsible for the study concept and design, modification of the study design, review and interpretation of the data, contributed to the collection and analysis of data, contributed to the interpretation of the data, and revised the manuscript. T-CF and C-MC were responsible for drafting the manuscript. Y-YK, J-RL, and C-MC made modifications to the study design and revised the manuscript. All authors read and approved the final manuscript.
